# Nailfold Videocapillaroscopy in Patients with Rheumatoid Arthritis and Psoriatic Arthropathy on ANTI-TNF-ALPHA Therapy

**DOI:** 10.3390/diagnostics13122079

**Published:** 2023-06-15

**Authors:** Daniela Anghel, Carmen Adella Sîrbu, Oana-Georgiana Petrache, Daniela Opriș-Belinski, Maria Magdalena Negru, Violeta-Claudia Bojincă, Cristina Florentina Pleșa, Florentina Ioniță Radu

**Affiliations:** 1 Department of Internal Medicine, ‘Dr. Carol Davila’ Central Military Emergency University Hospital, 010242 Bucharest, Romania; 2 Department of Medico Surgical and Prophylactic Disciplines, Titu Maiorescu University, 031593 Bucharest, Romania; 3 Department of Neurology, ‘Carol Davila’ University of Medicine and Pharmacy, 020021 Bucharest, Romania; 4 Department of Neurology, ‘Dr. Carol Davila’ Central Military Emergency University Hospital, 010242 Bucharest, Romania; 5 Department of Internal Medicine and Rheumatology, ‘Carol Davila’ University of Medicine and Pharmacy, 020021 Bucharest, Romania; 6 Department of Rheumatology, ‘Sf. Maria’ Clinical Hospital, 011172 Bucharest, Romania; 7 Department of Internal Medicine, ‘Sf. Maria’ Clinical Hospital, 011172 Bucharest, Romania; 8 Department of Internal Medicine and Gastroenterology, ‘Carol Davila’ University of Medicine and Pharmacy, 020021 Bucharest, Romania; 9 Department of Gastroenterology, ‘Dr. Carol Davila’ Central Military Emergency University Hospital, 010242 Bucharest, Romania

**Keywords:** nailfold capillaroscopy, rheumatoid arthritis, psoriatic arthritis, anti-TNF-alpha therapy, angiogenesis

## Abstract

Videocapillaroscopy is a simple, non-invasive investigation that allows the “in vivo” study of the nailfold capillaries. This method is inexpensive, easily accepted by patients and the results can be easily interpreted. It is mainly used in patients with Raynaud’s phenomenon and systemic sclerosis, but this examination can also be performed on patients who are suspected of having microcirculation alterations, such as rheumatoid arthritis and psoriatic arthritis. It may aid in the diagnosis, evaluation and prognosis of other rheumatic diseases, besides systemic sclerosis. The aim of this study is to identify the nailfold videocapillaroscopic abnormalities in rheumatoid arthritis and psoriatic arthritis patients and analyze the correlation between their evolution and 12 months of anti-TNF-α therapy. The abnormal capillaroscopic findings comprised widened, dilated or giant capillaries and the distortion of the normal nailfold architecture, avascular areas, hemorrhages and neoangiogenesis. Overall, capillary density, dilated capillaries, giant capillaries, elongated capillaries and angiogenesis significantly improved after 12 months. Moreover, no avascular areas were found after 12 months of anti-TNF treatment.

## 1. Introduction

The morphological changes of the microcirculation can be found in all autoimmune rheumatic diseases, not only in Raynaud’s phenomenon and systemic sclerosis. Nailfold capillaroscopy is the most widespread method of examination of the microvascular morphological alterations. It is the gold standard investigation for the differential diagnosis between primary and secondary Raynaud’s phenomenon. There are several non-invasive tools that can be used, but videocapillaroscopy has been shown to yield the best results [1]. 

Endothelial cell dysfunction and microcirculation damage take place at an early stage in the pathogenesis of autoimmune rheumatic diseases. Patients with autoimmune diseases have an increased risk of cardiovascular events. Moreover, associated risk factors such as smoking, obesity, sedentary lifestyle and even air pollution can contribute [2]. 

Andrade et al. reported morphological anomalies in almost 35% of the healthy subjects. Minor morphological abnormalities such as tortuosity can be found in approximately 10–20% of healthy subjects (more frequently in smokers and after repeated microtrauma) [3].

Psoriatic arthritis is a chronic inflammatory spondylarthritis that occurs in 1/3 of patients with psoriasis. Additionally, psoriatic arthritis may occur in patients without skin lesions. This type is rare and can develop in 15% of patients [4].

This rheumatic disease can cause peripheral arthritis, enthesitis, dactylitis and sacroiliitis that can lead to bone erosions, the destruction of the joints and permanent deformities. There are no laboratory findings specific for psoriatic arthritis [5].

Microvascular changes are considered to play a major role in the pathogenesis of psoriasis. The microcirculation of patients with psoriasis is significantly altered compared to healthy individuals. The presence of abnormalities such as edematous dermal and epidermal papillae or dilated and coiled capillaries can be visible in these patients. It has been shown that vascular changes precede skin lesions [6,7].

Studies have found that psoriatic arthritis patients have a decreased capillary density, dilated venular branches, tortuous and coiled capillaries and lower mean length and meandering capillaries with tight terminal convolutions and visible subpapillary venous plexus. Moreover, avascular areas were significantly more frequent in patients with psoriatic nails. This may suggest trophic disruptions in the periungual area of these subjects [8,9].

Rheumatoid arthritis is a rheumatic autoimmune disease that causes not only joint destruction but also vascular (microvascular and macrovascular) damage, thus increasing morbidity and mortality. Microvascular involvement is present from the early stage of RA and can be seen even in the absence of macrovascular damage. Rheumatoid vasculitis, a serious complication of rheumatoid arthritis, can occur in severe forms of the disease [10].

Nailfold capillaroscopy is one of the best imaging techniques that allows the “in vivo” evaluation of the microcirculation. This technique has shown increasing relevance not only for rheumatic diseases but also in the study of arterial hypertension, cardiac syndrome X, diabetes mellitus, venous insufficiency, etc. [11].

A videocapillaroscope combines a microscope that has a high magnification capability with a video camera. Precise measurements can be performed using the software.

Tumor necrosis factor alpha is a pleiotropic cytokine involved in the pathogenesis of several inflammatory and autoimmune diseases. It is considered the main pro-inflammatory cytokine involved in the pathogeny of rheumatoid arthritis, but it also plays an important role in psoriatic arthritis [12].

In rheumatoid arthritis, TNF-α is known as the main inflammatory cytokine and has been found in high concentrations in patients’ serum. T helper cells 1 (Th1) and macrophages are the major inflammatory cells that secrete TNF-α. This cytokine further activates fibroblasts, stimulates epidermal hyperplasia, recruits inflammatory cells and contributes to angiogenesis and osteoclasts activation. IL-1 and IL-6 also play an important role in joint erosion. Fibroblasts, activated by these cytokines, start producing cathepsins and matrix metalloproteinases (MMPs) that break down collagen and proteoglycans [13,14].

In psoriatic arthritis, inflammation is induced by Th1 and Th17 pathways. Th1 cell differentiation and propagation leads to the production of pro-inflammatory cytokines, TNF-α being one of them. TNF-α promotes erosions through osteoblasts inhibition and osteoclasts promotion [15].

TNF-α inhibitors are drugs that help lower TNF-α levels and therefore reduce inflammation in chronic rheumatic diseases. Anti-TNF-α agents have been approved in the treatment of RA and PsA. Etanercept is a recombinant soluble human TNF receptor p75Fc fusion protein which blocks TNF receptors. Adalimumab is the first fully human IgG1 monoclonal antibody and specifically targets and blocks TNF-α. Infliximab is a IgG1κ monoclonal antibody that targets the soluble and transmembrane forms of TNF- α with high affinity. It also prevents TNF-α from binding to its receptor [16].

The aim of this study is to identify the nailfold videocapillaroscopic abnormalities in rheumatoid arthritis and psoriatic arthritis patients and analyze the correlation between their evolution and 12 months of anti-TNF-α therapy.

## 2. Materials and Methods

### 2.1. Subjects

We performed a retrospective observational study on 92 male and female patients who were diagnosed with rheumatoid arthritis (and met EULAR/ACR2010 criteria) and patients with psoriatic arthritis (who met CASPAR criteria) who were administered anti-TNF-alpha therapy.

Our study aimed to observe the capillaroscopic abnormalities of these patients before the treatment with anti-TNF-alpha and after 12 months of therapy. The study was conducted in one center (“Carol Davila” Central Emergency Military University Hospital, Bucharest, Department of Internal Medicine 2), between September 2021 and September 2022. 

We divided the patients into approximately 3 groups: the rheumatoid arthritis group, comprising 34 patients; the psoriatic arthritis group, composed of 34 patients; and the control group, made up of 24 patients.

Exclusion criteria: 1. Patients with clinical manifestations of atherosclerosis such as coronary artery disease, peripheral vascular disease and cerebrovascular disease. 2. Age under 18 years. 3. History of smoking or alcoholism. 4. Patients with constant professional trauma of the fingers or active visible trauma/cosmetic procedures. 5. Patients with history of biological treatment.

This study was approved by the local ethics committee and all subjects gave written informed consent.

### 2.2. Examination Method

In order to examine the microcirculation in rheumatoid and psoriatic arthritis patients, we used a videocapillaroscope. The examination site was the nailfold area, where capillaries are parallel to the skin surface and not perpendicular as in other skin surfaces. The capillaries can be observed in their full length in the last row. 

Before the examination, patients stayed at room temperature of 20–24 °C for 15 min. They were asked to avoid smoking and drinking caffeinated beverages for 4 h prior the examination. Additionally, they were asked not to undergo cosmetic procedures of the nails in the previous two weeks or remove fingernail cuticles for one month in order to avoid potential false-positive results. In order to improve resolution, because of its higher viscosity, one small drop of cedar oil was used on the nailfold area. The capillaries could be seen at different magnifications, but we used 200x, the preferable magnification. The videocapillaroscope touched the patient’s nailfold directly, but it did not press the patient’s skin because this could have modified the vessels. Reflections were minimized by changing the angle of the videocapillaroscope. All fingers were evaluated, but measurements were performed only on the fourth and fifth digits where the skin is more transparent and capillaries can be seen more clearly. In order to prevent vasodilation, a cold light source was used. 

### 2.3. Investigated Parameters

Standard parameters, such as capillary density, length, width, architectural structure, tortuosity, the presence of angiogenesis, microhemorrhages and subpapillary venous plexus visibility, were analyzed. 

A normal pattern consists of “hairpin shape/reverse U-shape” capillaries that are homogenous with small morphological variations. Capillary disorganization is defined as the complete distortion of the standard capillary pattern. Usually, there are 1–3 capillaries in each dermal papilla. The capillary has one arterial limb (afferent) and one venous limb (efferent). The afferent limb width (the widest section of a capillary loop) varies from 7–17 µm and from 11–20 µm in the efferent limb. The ratio between the efferent and afferent loop should not exceed 2:1.

**Scheme 1:**
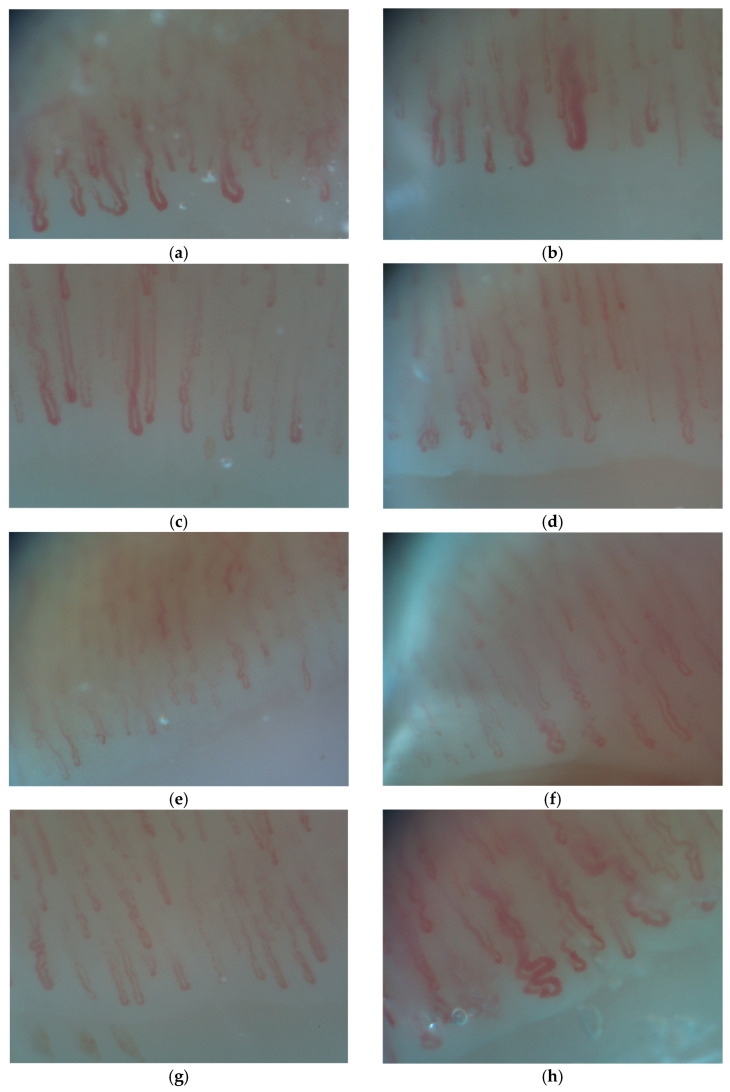
(**a**) Dilated capillaries in PsA patient; (**b**) Megacapillary in a PsA patient; (**c**) Elongated capillaries in a PsA patient; (**d**) Avascular area in a PsA patient; (**e**) Tortuous capillaries in a RA patient; (**f**) Crossed capillaries in a RA patient; (**g**) Hemorrhages in a RA patient; (**h**) Angiogenesis in a PsA patient.

There are 3 types of enlarged capillary loops: enlarged efferent, enlarged afferent and enlarged apical capillaries. If the capillary loop width exceeds 20 µm, the capillary is considered enlarged. Capillaries with a limb diameter >17 µm or a venous limb wider than 20 µm are considered dilated capillaries. A width exceeding 50 µm defines a giant or megacapillary (Scheme 1a,b) [17].

The length of the capillary varies from person to person and is usually between 200–300 µm. If the capillary loops are longer than 300 µm, they are considered elongated. (Scheme 1c) 

The parameters (width and length) can be measured manually or using the automated software of the videocapillaroscope. Some important points should be taken into consideration: capillary width is age-related, and the capillary length of the 4th and 5th digits are longer than those of the other fingers [18,19,20].

Capillary density is defined as the number of capillaries in 1 mm length of the distal row. The average capillary density is considered ≥7 per 1 mm. If the number of loops is lower than 7, the density is considered low. We used the direct observation method (the distal loops are observed and marked) for measuring the number of capillaries.

Avascular areas are defined as areas with a distance between two adjacent capillary loops of greater than 500 µm (Scheme 1d).

Tortuous capillaries present multiple curves but do not cross over themselves. Meandering capillaries are capillaries which have limbs that intersect at ≥2 points with themselves or with other capillaries (Scheme 1e). Crossed capillaries have limbs that cross over each other (Scheme 1f). Ramified and bushy capillaries are capillaries that lose their “hairpin shape” and branch out.

Hemorrhage is defined as extracapillary blood or hemosiderin deposits at a variable distance from the distal row due to the rupture of the capillary wall and blood extravasation (Scheme 1g). 

The subpapillary venous plexus (in which the efferent limb incorporates) can be seen in almost 30% of the healthy individuals, but it is more frequently visualized in patients with rheumatoid arthritis [21].

Angiogenesis (the formation of new capillaries after capillary loss) is present if the capillaries are extremely tortuous or “bushy/coiled”, if a dermal papilla holds more than 4 capillaries or if the capillaries have extremely elongated loops, are branched, thin and interconnected (Scheme 1h). Angiogenesis dysregulation has been detected in the synovial tissues and serum of patients with autoimmune rheumatic diseases. The pathogenic mechanisms of this process are still unclear. Most likely, an imbalance between antiangiogenic and angiogenic factors is the starting point of the deregulated angiogenesis [22].

It is known that TNF-alpha induces angiogenesis and some anti-TNF-alpha agents have been shown to inhibit angiogenesis and growth in vivo, in oral squamous carcinoma cells [23].

Synovial arthroscopic biopsies performed on rheumatoid arthritis patients have found a large amount of new, undeveloped blood vessels [24].

TNF-alpha inhibitors are also involved in the reduction in vascular endothelial growth factor (VEGF) levels in the serum of psoriatic arthritis patients [25].

The subpapillary plexus is composed of multiple blood vessels and can be visible in young children. It becomes progressively more invisible with age, but in people with more transparent skin, as elderly people, the subpapillary venous plexus can be visible [26].

The same technician acquired all the images before and after 12 months of treatment.

The disease activity score 28 (DAS 28), a score that measures the activity of the disease in RA patients, was used. Severe activity is defined as a DAS 28 of > 5,1, moderate activity as 2.6–3.2 and a low activity disease as having a score of <3.2. The disease activity in PsA patients was measured with DAPSA (disease activity index for psoriatic arthritis). A score of ≤4 is defined as remission, >4 and ≤14 as low disease activity, a score of >14 and ≤28 as moderate disease activity and a score of >28 as high disease activity.

### 2.4. Statistical Analysis

Statistical analysis was performed with SPSS (statistical program for social science) software (SPSS Version 11 Inc., Chicago, IL, USA) as follows: the description of quantitative variables as mean, standard deviation (SD), median and range. Pearson’s correlation coefficient value of 0.01 was considered to reflect a high statistical significance. A value lower than 0.05 is considered statistically significant. A value higher than 0.05 is considered non-significant. To compare parameter evolution after 12 months, we used Friedman’s ANOVA and one-way repeated measures ANOVA tests. The correlation matrix was also used as a tool to summarize a large dataset as follows: −1 suggests a perfectly negative linear correlation between two variables, 0 suggests no correlation and 1 suggests a perfectly positive linear correlation.

## 3. Results 

We analyzed data from patients before the use of anti-TNF therapy (baseline) and at follow-up after 12 months of treatment (standard evaluation time). 

In the rheumatoid arthritis group, 34 patients were included. All these patients received TNF-alpha inhibitors: Etanercept (41.2%), Infliximab (26.5%) and Adalimumab (32.3%).

Patients were grouped according to their sex and age. A total of 26.5% were between 20 to 40 years old, 44.1% were between 41 to 60 years old and 29.4% were over 60 years old. The women to men ratio was 2:7.

Structural integrity was observed in only 41.2% of patients at baseline and 64.7% in anti-TNF treated patients. 

Baseline capillaroscopic density was low in 32.4% of rheumatoid arthritis patients. After 12 months of treatment, low capillary density was found in only 11.8% of patients. Avascular areas were found in 20.6% of the subjects with rheumatoid arthritis at baseline and in 0% of the patients after 12 months of anti-TNF-alpha treatment. 

Patients with elongated capillaries were found in 20.6% of the rheumatoid arthritis group at baseline and only in 14.7% of the patients after they received 12 months of treatment.

Tortuous capillaries were present in 70.6% of patients at baseline and in 38.2% of patients after 12 months. Bushy capillaries were present in 47.1% of patients at baseline and in 11.7% after 12 months of treatment. Meandering capillaries were found in 52.9% of patients at baseline and in 50.6% of patients after treatment. Neoangiogenesis was present in 52.9% of patients at baseline and in none of the patients after 12 months of treatment. 

Dilated capillaries were found in 61.8% of rheumatoid arthritis patients at baseline and 29.4% after 12 months. We observed that 35.3% of patients had giant capillaries at baseline and only 14.7% of them had after 12 months.

Microhemorrhages were observed in 23.5% of patients at baseline and in 0% of the patients after 12 months of TNF-inhibitors. 

Regarding the use of type anti-TNF agents, no notable differences were found between the groups. Additionally, there was no correlations between patient’s gender or age and their capillaroscopic patterns. 

Altered microvascular architecture was significantly more frequent in patients with a scleroderma-like pattern than patients with a normal pattern (*p* < 0.05). Moreover, angiogenesis, isolated enlarged loop, irregular enlarged loop and architectural derangement were significantly higher in patients with a scleroderma-like pattern in comparison to patients with a normal pattern (*p* < 0.05).

In the psoriatic arthritis group, 33 patients were included, who were treated with TNF-alpha inhibitors as follows: Etanercept (48.5%), Infliximab (24.2%) and Adalimumab (27.3%). The group was divided into three age categories: 20–40 years old (17.6% of patients), 40–60 years old (58.8% of patients) and >60 years old (23.5% of patients).

We observed that 54.5% of these patients were females, and 45.5% of them were males.

Structural integrity was detected in only 29.4% of the patients at baseline and in 67.6% of them after 12 months of anti-TNF therapy.

Low density was found in 30.3% of the patients at baseline and in 15.2% of the patients after 12 months of TNF inhibitors. Avascular areas were present in 39.4% of the patients and in only 24.2% of the patients who received treatment. 

Elongated capillaries were found in 57.5% of the rheumatoid arthritis patients at baseline and only in 42.42% of the patients after they received treatment. Tortuous capillaries were present in 54.5% of the subjects at baseline and in only 21.2% of the subjects after treatment. Meandering capillaries were found in 15.2% of the patients at the beginning of the study and in 15.2% after 12 months of therapy. Bushy capillaries were present in 21.2% of patients at baseline and in 21.2% of them after 12 months.

Neoangiogenesis was present in 48.5% of the subjects and in only 15.2% of the subjects who received treatment.

Dilated capillaries were found in 42.4% of the patients at baseline and in 0% of the patients after 12 months. Hemorrhages were present in 45.5% of patients at baseline and in 0% of patients after 12 months.

After 12 months of TNF-α, we observed that 100% of patients were in remission (DAS 28 < 3.2), 0% had moderate activity (DAS 28 = 3.2–5.1) and 0% had an active disease (DAS 28 > 5.1).

After 12 months of treatment, we observed a decrease in the number of giant capillaries (75.8% of patients), elongated capillaries (57.6% of patients), tortuous capillaries (54.5% of patients) and neoangiogenesis (48.5% of patients). Significant improvements were also noticed in hemorrhages (45.5% of patients), dilated capillaries (42.4%) and avascular areas (39.4%). 

In the control group, 24 subjects aged between 20–40 years old (25%), 41–60 years old (58.3%) and above 60 years old (16.7%) were included. In this group, 54.2% were female and 45.8% were male. None of the subjects had low capillary density or avascular areas. In the control group we found tortuous capillaries in 20.8% of times, meandering capillaries in 25% of patients, bushy capillaries in 33.3% of patients and dilated capillaries in 12.5% of the subjects, but none of them had giant capillaries or hemorrhages. Additionally, none of the subjects had neoangiogenesis.

**Figure 1:**
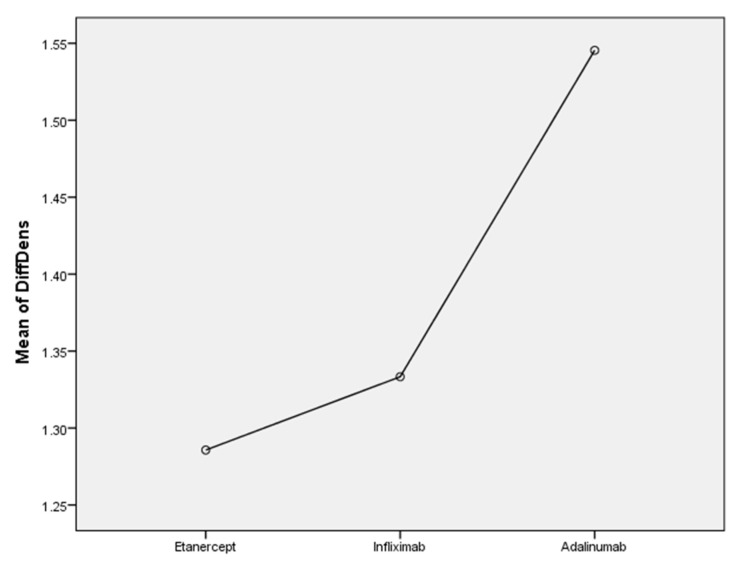
The efficacity of TNF-alfa inhibitors on capillary density in RA patients.

In order to compare TNF inhibitors, we divided the RA patients into 3 groups. All three groups had similar favorable results regarding capillary density (Figure 1). Regarding dilated capillaries, improvements were observed in 35.7% of patients treated with Etanercept, 55.5% of patients treated with Infliximab and only 9.1% of patients treated with Adalimumab. Regarding hemorrhages, patients treated with Adalimumab had the greatest improvements (54.5% of patients) after 12 months of treatment.

**Figure 2:**
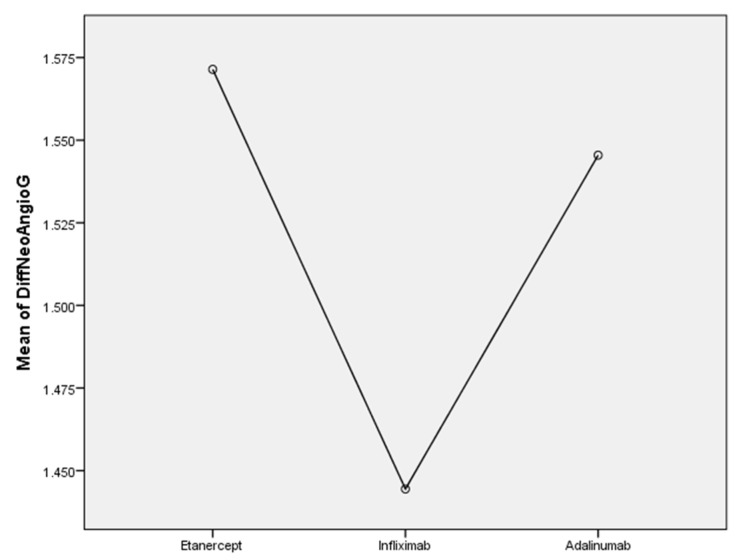
The efficacity of TNF-alfa inhibitors on neoangiogenesis in RA patients.

In the Etanercept group, we observed an improvement in neoangiogenesis (57.4%), Figure 2, and tortuosity (57.14%) patterns. In the Infliximab group, we found a reduction in neoangiogenesis (44.4%), dilated capillaries (55.5%) and crossed capillaries (44.4%). In the Adalimumab group, neoangiogenesis (54.5%), hemorrhages (54.5%) and bushy capillaries (45.4%) were reduced after 12 months of treatment.

**Figure 3:**
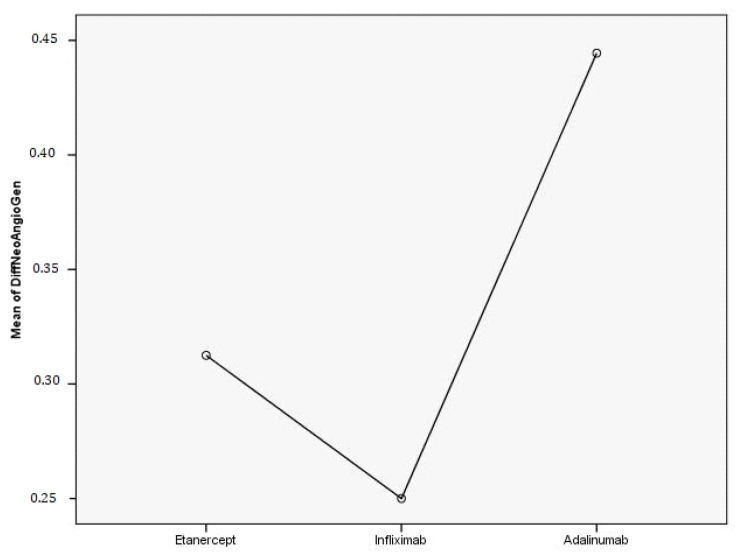
The efficacity of TNF-alfa inhibitors on neoangiogenesis in PsA patients.

In the psoriatic arthritis group, we observed a decrease in the number of giant capillaries (75.8% of patients), elongated capillaries (57.6% of patients), tortuous capillaries (54.5% of patients) and neoangiogenesis (48.5% of patients)—Figure 3. Significant improvements were also noticed in hemorrhages (45.5% of patients), dilated capillaries (42.4%) and avascular areas (39.4%). 

**Figure 4:**
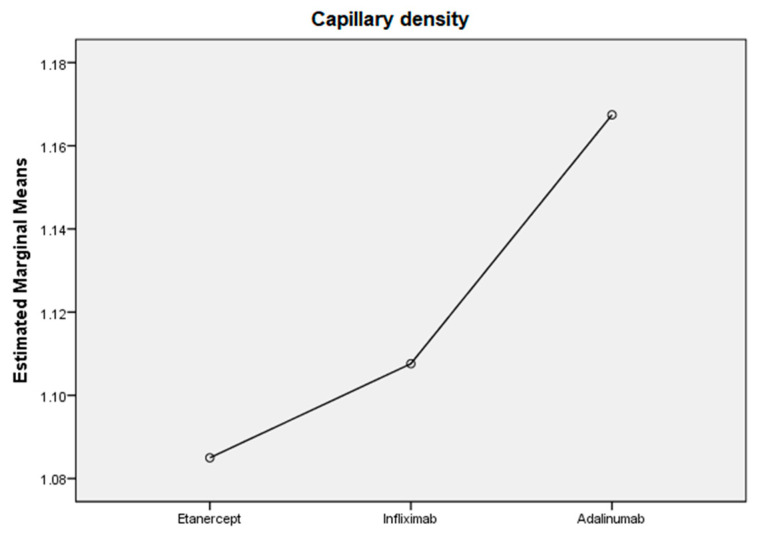
The efficacity of TNF-alfa inhibitors on capillary density in PsA patients.

Regarding the TNF inhibitor used, we divided the PsA group into three groups. In the Etanercept group, we observed an important reduction in the number of dilated capillaries (43.8% of patients), tortuous capillaries (37.5%), giant capillaries (37.5%) and hemorrhages (37.5%). In the Infliximab group, we observed reduced hemorrhages (62.5%) and a reduced number of giant capillaries (50%). In the Adalimumab group, we found an important reduction in the number of dilated capillaries (55.6%), giant capillaries (55.6%) and avascular areas (55.6%). In the Adalimumab group we observed a siginificant increase in capillary density (Figure 4).

Overall, we observed the following changes in capillary morphology at 12 months. A significant correlation between the use of anti-TNF agents and normal capillary density (*p* = 0.0001) was found. Additionally, we found a significant correlation between the decrease in avascular areas (*p* = 0.00018) and the decrease in dilated capillaries (*p* = 0.0001) after 12 months of anti-TNF agent use. We found a positive statistical correlation between the use of anti-TNF treatment and the decrease in the number of giant capillaries (*p* = 0.007), angiogenesis (*p* = 0.0003) and elongated capillaries (*p* = 0.0003). No correlations were found between the overall use of TNF inhibitors and the number of crossed (*p* = 0.69), bushy (*p* = 0.168) or tortuous capillaries (*p* = 0.19).

## 4. Discussion

Nailfold capillaroscopy can recognize peripheral microangiopathy, even in the early stages of autoimmune arthritis. Differential diagnosis in early arthritis can be challenging in PsA patients that present with mild or no cutaneous signs and in PsA patients with symmetrical joint involvement. The presence of a specific microvascular pattern may be an additional tool for differential diagnosis between RA and PsA. The use of anti-TNF therapy in patients with RA and PsA can reduce the incidence of nailfold capillaroscopic abnormalities observed in these diseases.

Compared to psoriatic arthritis, the main abnormalities found in rheumatoid arthritis patients were tortuous, dilated capillaries and the presence of neoangiogenesis and crossed capillaries. These findings have been mentioned in several studies regarding nailfold videocapillaroscopy in RA and PsA patients [27].

Other studies found non-specific capillaroscopic abnormalities, such as hemorrhages and the presence of venous plexus. RA patients present with a non-scleroderma pattern, with minor anomalies, such as tortuosity and capillary crossing [28,29].

A scleroderma-like pattern can be found in a small subgroup of patients with rheumatic diseases, other than systemic sclerosis. In our study, we found both RA and PsA patients with a scleroderma-like pattern (giant capillaries, hemorrhages, avascular areas or neoangiogenesis). These abnormalities, although non-specific, require closer monitoring [30,31].

Videocapillaroscopy in psoriatic arthritis patients found more giant capillaries and more elongated capillaries than in RA patients. Regarding capillary width, giant capillaries were found in more psoriatic arthritis patients than rheumatoid arthritis patients. Additionally, hemorrhages and avascular areas were observed in more PsA patients than RA patients. Regarding capillary density, we found no notable difference between the two groups at T0 (baseline). However, a lower density in PsA patients was previously observed by Graceffa et al., who performed capillaroscopy on 30 RA and 30 PsA patients. Faggioli et al. also found a lower density in PsA patients but found that tortuous capillaries were more prevalent in PsA compared to RA patients [8,32].

Angiogenesis plays a key role in the pathogenesis of psoriasis. Studies show that TNF-alpha contributes to angiogenesis. The histological hallmarks of the psoriatic skin lesions are dilated and elongated capillaries in the dermal papillae. The return to the normal state of the microvascular dermal plexus precedes the normalization of the epidermis [33].

A study performed on 99 rheumatoid arthritis patients observed a negative correlation between capillary density and CRP levels [34]. No correlation was observed in our study regarding this statement.

Anti-TNF-α agents have shown great therapeutic results both in RA and PsA patients.

Regarding the use of a specific anti-TNF-alpha agent (Etanercept, Infliximab, Adalimumab), some differences were observed between the groups as follows: In the RA group, Etanercept-treated patients saw the best improvement in tortuosity, Infliximab showed the most significant effect on dilated capillaries and both Adalimumab and Etanercept resulted in the improvement of neoangiogenesis. Regarding elongated capillaries, no major improvements were observed in either of the three groups. In the PsA group, Adalimumab-treated patients had the best improvement in dilated capillaries and avascular areas, while Infliximab showed the best results in hemorrhages. 

Nailfold videocapillaroscopy is un underused tool in the evaluation of other autoimmune diseases besides systemic sclerosis. In addition to its potential role in the differential diagnosis of early arthritis, it can be used to evaluate the severity of the disease and to determine the efficacy of the therapy. Another study has concluded that capillaroscopic abnormalities can predict patients’ long-term prognosis [35]. Regarding this statement, no correlations were made. A longitudinal study on a large number of patients is needed in order to evaluate the relationship between capillaroscopic abnormalities and patient’s long-term prognosis.

This method has become more relevant in recent years in systemic sclerosis, but studies have shown its importance even in inflammatory myositis, in interstitial lung disease and even in other medical specialties (cardiology) [36].

## 5. Conclusions

In conclusion, according to our findings, after 12 months of anti-TNF-alpha therapy, capillaroscopic abnormalities, such as neoangiogenesis, dilated/giant capillaries and avascular areas, improved. No significant changes were observed in parameters such as tortuous, crossed or bushy capillaries.

Capillaroscopic abnormalities and their clinical significance in rheumatoid arthritis and psoriatic arthritis remain the subject of an ongoing debate. Nailfold capillaroscopy could be a useful tool in the differential diagnosis of early arthritis, in the evaluation of the disease severity and in monitoring the efficacy of biological treatment in patients with inflammatory arthritis.

Our study had the following limitations: a limited number of patients. A larger sample size will provide more information about videocapillaroscopic abnormalities in RA and PsA patients on anti-TNF-α therapy.

## Data Availability

Any materials or data that are reasonably requested can be provided in a timely fashion to members of the scientific community for noncommercial purposes.
